# The vertebrate muscle superlattice: discovery, consequences, and link to geometric frustration

**DOI:** 10.1007/s10974-023-09642-8

**Published:** 2023-05-13

**Authors:** Rick P. Millane, Pradeep K. Luther

**Affiliations:** 1 Computational Imaging Group, Department of Electrical and Computer Engineering, University of Canterbury, Private Bag 4800, Christchurch, New Zealand; 2 Cardiac Function Section, National Heart and Lung Institute, Imperial College London, Hammersmith Campus, ICTEM Building, Du Cane Road, W12 0NN London, UK

**Keywords:** Vertebrate muscle, Myosin superlattice, Frustration, Ising model

## Abstract

Early x-ray diffraction studies of muscle revealed spacings larger than the basic thick filament lattice spacing and led to a number of speculations on the mutual rotations of the filaments in the myosin lattice. The nature of the arrangements of the filaments was resolved by John Squire and Pradeep Luther using careful electron microscopy and image analysis. The intriguing disorder in the rotations, that they termed the myosin superlattice, remained a curiosity, until work with Rick Millane and colleagues showed a connection to “geometric frustration,” a well-known phenomenon in statistical and condensed matter physics. In this review, we describe how this connection gives a satisfying physical basis for the myosin superlattice, and how recent work has shown relationships to muscle mechanical behaviour.

## Introduction

In this review, we celebrate the role of our esteemed friend John Squire in highlighting the occurrence, and solving the nature, of the myosin superlattice in vertebrate striated muscles, and describe how we linked the finding to a fundamental phenomenon in physics. We review this journey and provide some new insight into the nature of the superlattice. 

**Fig. 1:**
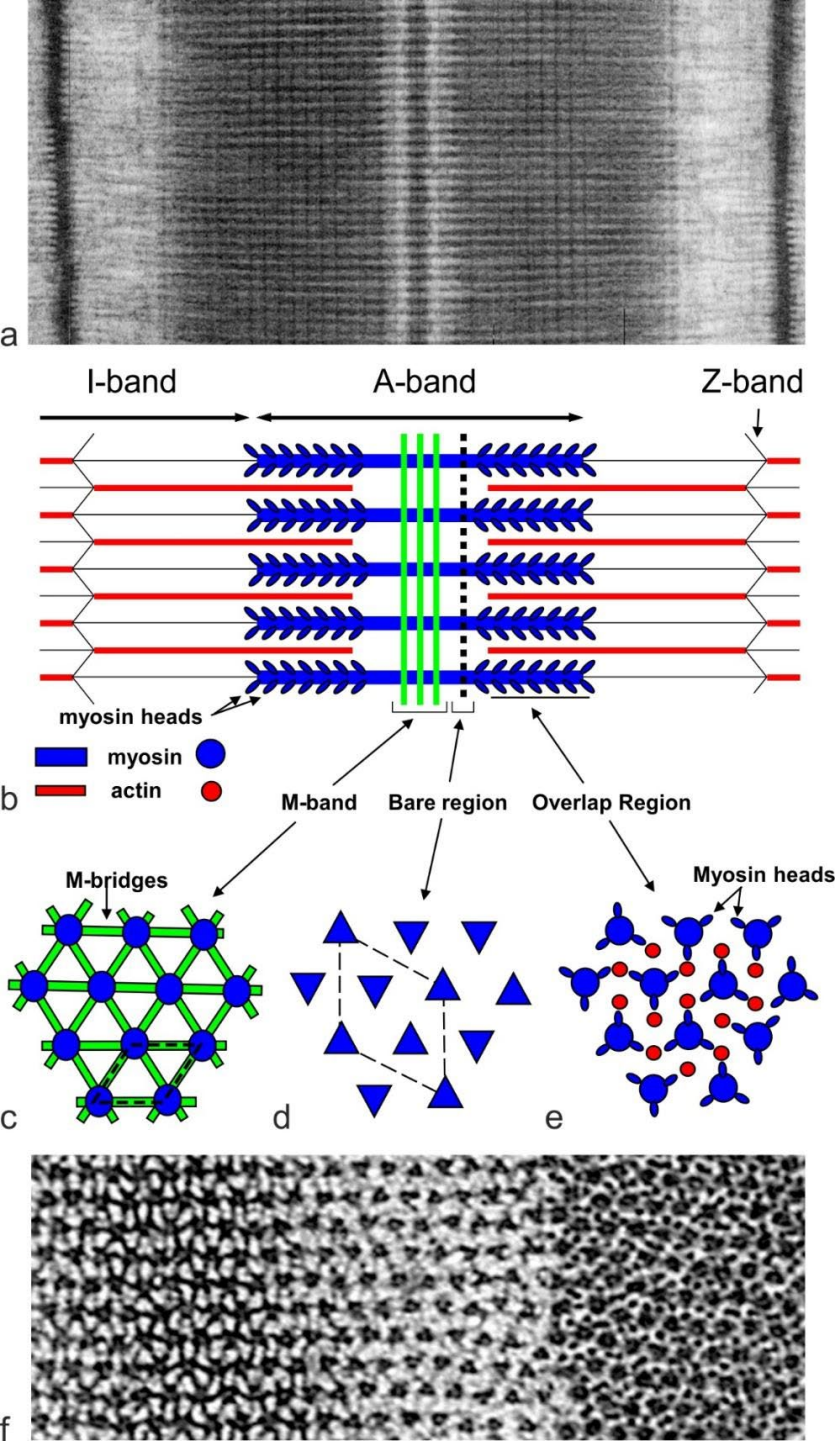
Vertebrate striated muscle sarcomere and the myosin filament superlattice. (a) Electron micrograph of frog sartorius muscle sarcomere and (b) schematic diagram of the sarcomere. The sarcomere is bounded by the Z-bands from which emanate actin filaments which overlap with the myosin filaments in the A-band. Contraction of muscle occurs when crossbridges on the myosin filaments interact with actin filaments to bring them closer to the centre of the A-band. The bipolar myosin filaments are crosslinked at the centre by the M-band assembly (b,c) which determines their relative rotation about the long axis. (c,d,e) Show schematic views of cross-sections in the M-band, bare region and the crossbridge region, respectively. (f) Shows electron micrographs of transverse sections through the M-band (left), bare region (centre) and crossbridge region (right). In the crossbridge region, the filament cross-section profile is indistinct hence the axial rotations of the filaments cannot be determined. In the bare region between the A-band and the start of the crossbridge region, the myosin filaments have a triangular profile (d and f centre) and this enables determination of the axial rotations of the filaments within 60^o^. In a hexagonal lattice we can define a simple unit cell (c, dashed outline). In certain vertebrate muscles, the myosin filaments tend to have identical orientations for second-next nearest neighbours (d) and so are arranged on a superlattice (dashed outline). Parts b,c,d,e adapted from Millane et al. (2021)

Striated muscles in vertebrates are impeccably ordered, giving beautiful patterns at different scales. Fibres viewed under a high-power light microscope display fine ~ 2 μm striations over the whole length of the fibre. Under an electron microscope, the striations are revealed as sarcomeres joined end to end via the Z-lines (Fig. 1a, b). Actin filaments emanate from each side of the Z-line and overlap with the myosin filaments in the A-band. The myosin filaments have crossbridges over most of their length, and the crossbridges interact with actin filaments to increase the length of their overlap, producing contraction of the muscle. Myosin filaments are bipolar and at their centre are tethered by the M-band assembly into a hexagonal lattice, which also determines their axial rotations.

**Fig. 2:**
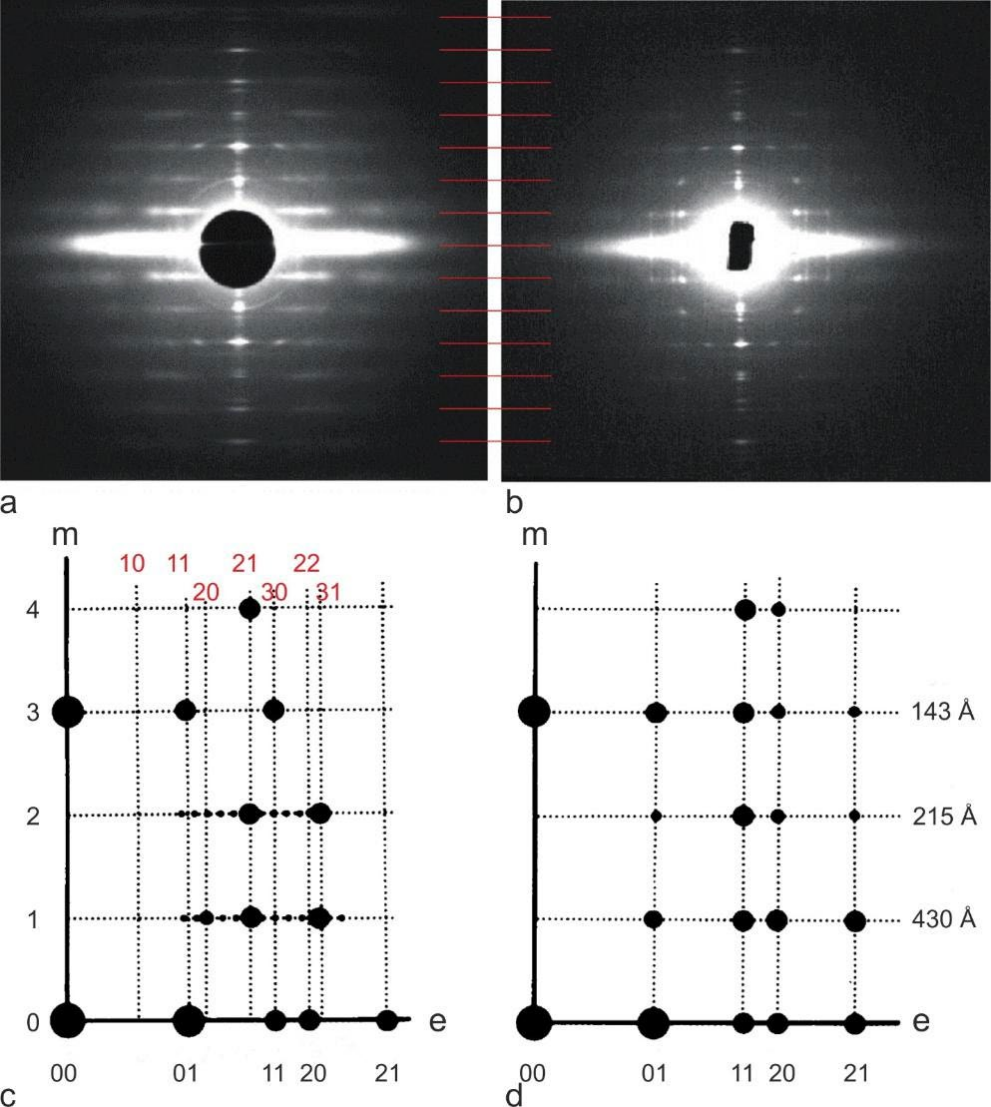
X-ray diffraction patterns of frog skeletal muscle (superlattice, a), and fish skeletal muscle (simple lattice, b), and diagrams of their main features (c,d). Muscle fibres are mounted vertically. The helical order of the crossbridges is revealed by reflections on the meridian (m) and along the layer lines (labelled 1,2, etc. in c) which are orders of the 430 Å repeat of the myosin crossbridges. Perpendicular to the fibre direction is the equator (e) comprising reflections from the hexagonal lattice of the filaments (indices labelled 01, 11 etc. in black in c and d). Huxley and Brown (1967) found that reflections on the 1st and 2nd layers lines in some muscles (frog in the case shown here) index on a superlattice which is $\surd$3 times larger than the simple (basic) hexagonal lattice (indices 10, 11 etc. labelled in red in c). (Adapted from Harford and Squire (1986)).

Since vertebrate striated muscle is highly ordered, small angle x-ray diffraction has provided much insight into its ultrastructure, although it does not give actual images (e.g. Figure 2a, b), and gives information on live muscle without any processing steps. Electron microscopy (EM), on the other hand, gives direct images, although the various chemical processing steps required means that the internal structure of the sample may be altered. However, when used together, periodicities of live muscle derived from x-ray diffraction can help to confirm the structural features observed by EM. The pioneer of x-ray diffraction of muscle was Hugh Huxley with his classic tour de force study in 1967 (Huxley and Brown 1967). They noticed that for frog muscle, while the x-ray reflections on the equator (labelled e in Fig. 2c, d) are compatible with thick filaments based on a hexagonal lattice, diffraction spots on the 1st and 2nd layer lines (Fig. 2a, c) cannot be indexed on this simple lattice (Fig. 1c), but they can be indexed on a lattice √3 times larger (indices shown in red in Fig. 2c). This means that there are subtle differences between the axial rotations of neighbouring filaments. They suggested that the rotations are the same only for second-nearest neighbours, and the filaments are arranged on a superlattice √3 times larger than the regular lattice (Fig. 1c, d). This is in contrast to fish skeletal muscle which shows only the simple lattice reflections (Fig. 2b, d).

**Fig. 3:**
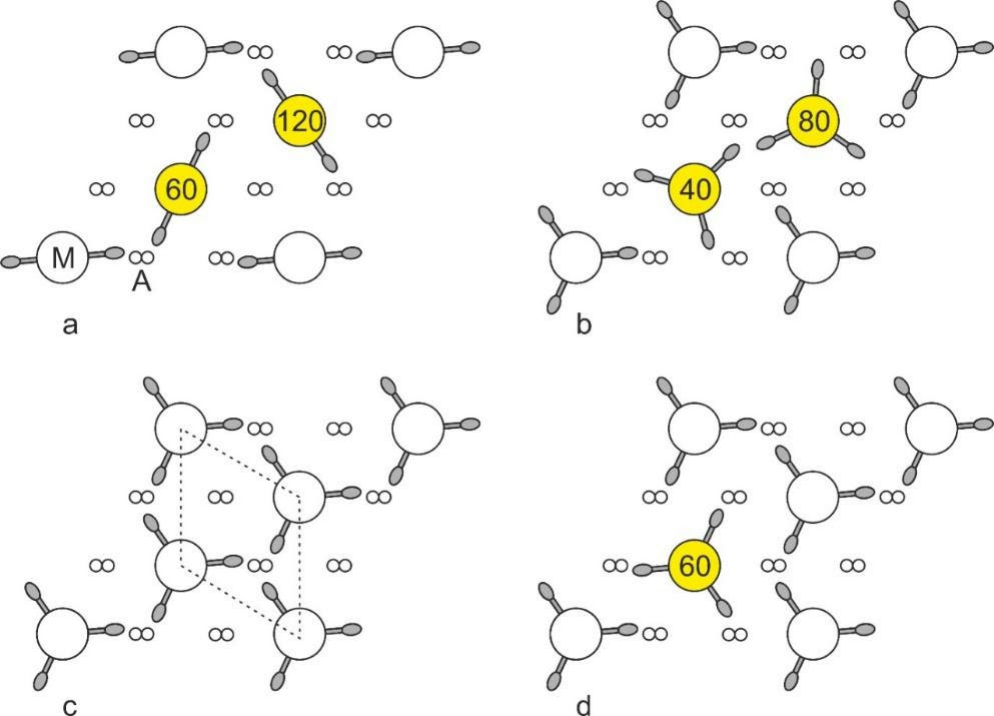
Crossbridge environments within a thin slice of vertebrate striated muscle within a single superlattice unit cell. M and A depict myosin and actin filaments, respectively. By definition, the rotations of the corner filaments of the superlattice unit cell are identical. Where the rotations are different to the corner rotations, they are coloured yellow. Initially, perfect superlattice arrangements were proposed (a and b). (a) For a 2-stranded myosin filament (where rotations of 0^o^ and 180^o^ are equivalent), Huxley and Brown (1967) proposed 60^o^ rotations between neighbouring filaments. (b) For a 3-stranded myosin filament, Squire suggested 40^o^ rotations between neighbouring filaments (Squire 1974). Perfect superlattice arrangements were not found however in electron micrographs, but the arrangement is a statistical superlattice (Luther and Squire 1980). Crossbridge arrangements for a simple lattice (c) and a superlattice muscle (d). (c) In simple lattice muscle (unit cell shown in dashed lines), all myosin filament rotations are identical and the actin filaments are approached by three or no crossbridges. In superlattice muscles (d), the two internal filaments are arranged statistically and the actin filaments are approached by one or two crossbridges

Huxley and Brown suggested that if the thick filaments are composed of myosin molecules packed in a 2-stranded fashion, then a perfect hexagonal superlattice can be generated if neighbouring filaments are mutually rotated by 60^o^, as shown in Fig. 3a. John showed that other arrangements are also possible (Squire 1974). Firstly, he proposed that the filaments may be 3 or 4-stranded. He proposed that for a 3-stranded filament a perfect superlattice could be achieved with 40^o^ mutual rotations between neighbours (Fig. 3b). In the end, as described below, neither John’s 40^o^ nor Huxley’s 60^o^ arrangements were found.

To understand how the superlattice is organised, direct imaging by EM was required. This was not an easy task, as cross-sections of thick filament show fuzzy outlines over most of their length (Fig. 1f). It was John’s sharp insight to notice that in the tiny ~ 50 nm “bare” region between the M-band and the start of the crossbridge region (Fig. 1b,f centre), the thick filaments have triangular profiles, allowing their rotations to be measured to within 60^o^. In 1974, he assigned this as the project for his first PhD student (one of the authors, PKL).

## Finding the nature of the superlattice by electron microscopy

**Fig. 4:**
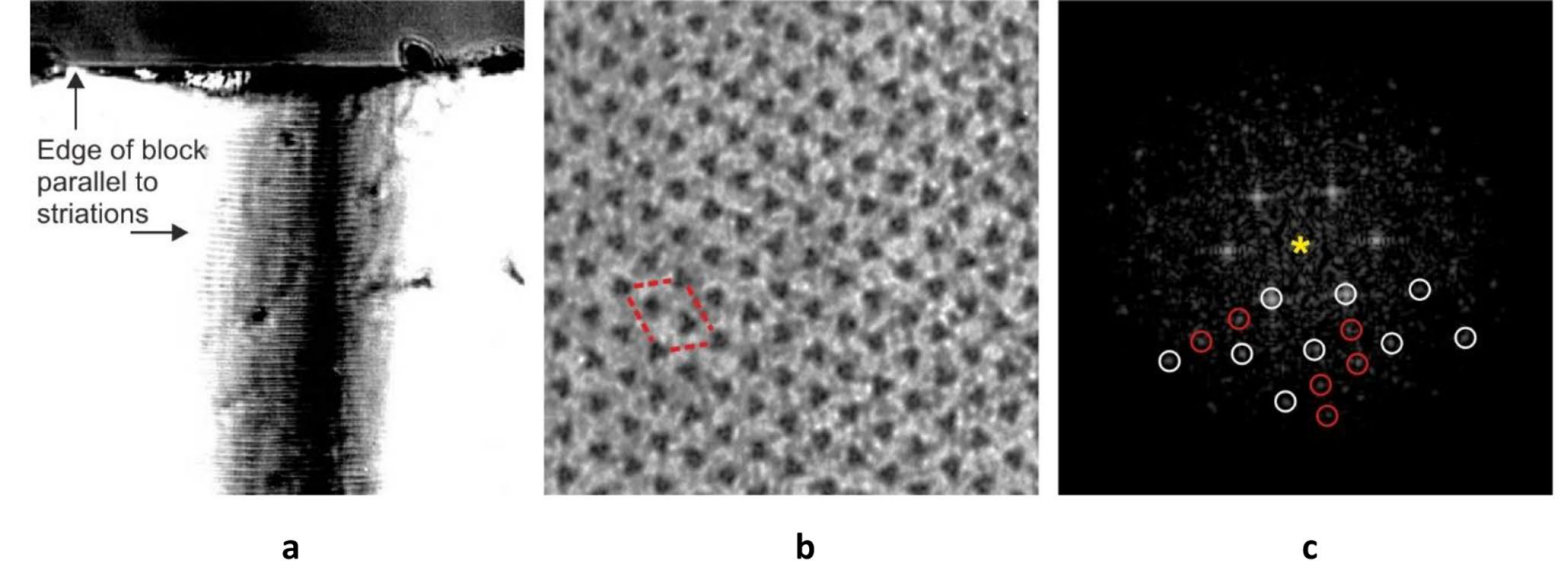
Preparation of “perfect” transverse sections. (a) To obtain almost perfectly oriented transverse sections, the block with face partially trimmed was removed from the microtome and observed under a microscope (preferably inverted) to view the block edge and the striations of the embedded muscle. The block was returned to the microtome and the block oriented to ensure that the block edge was parallel to the striations. (b) Transverse section of bare region of frog sartorius muscle. A superlattice unit cell is outlined. (c) Diffraction pattern (Fourier transform) of (b) shows simple lattice spots (white circles) and superlattice spots (red circles). The pattern is symmetrical about the centre (yellow star) and the circled spots can be compared to the unmarked spots in the upper half of the pattern

There were two obstacles to overcome in the project. Since the bare region has a small axial width of ~ 50 nm, a near perfect transverse section was required to obtain a reasonable area of bare region. To achieve this, the embedded muscle block was viewed with an inverted compound microscope to check the orientation of the sarcomere striations and the block face edge (Fig. 4a). Then, the block was rotated to make the face parallel to the striations (Fig. 4a). In native muscle, the myofibrils in a fibre are organised so that the Z-lines and M-bands are in register, however this is easily disrupted during EM processing. To improve the lateral register of the myofibrils, handling of the muscle was minimised by fixing the whole skinned leg in glutaraldehyde and then a superficial slice cut from the sartorius muscle followed by conventional processing. Perfecting the sample preparation involved many trials and took PKL nearly 2 years. This slow progress worried John immensely and near the end of this period, he started planning to abandon the superlattice EM project and start PKL on a new non-EM project.

Finally, however, ideal samples were prepared from which excellent EM images of transverse sections were obtained which showed extensive areas of bare region with marked triangular profile myosin filaments (Fig. 4b). The images also showed new details of the M-band that we reported in Luther and Squire (1978). Optical diffraction of the bare region electron micrographs showed beautiful, clear superlattice spots in addition to the simple (basic) lattice spots (red and white circles spots, respectively, in Fig. 4c).

**Fig. 5:**
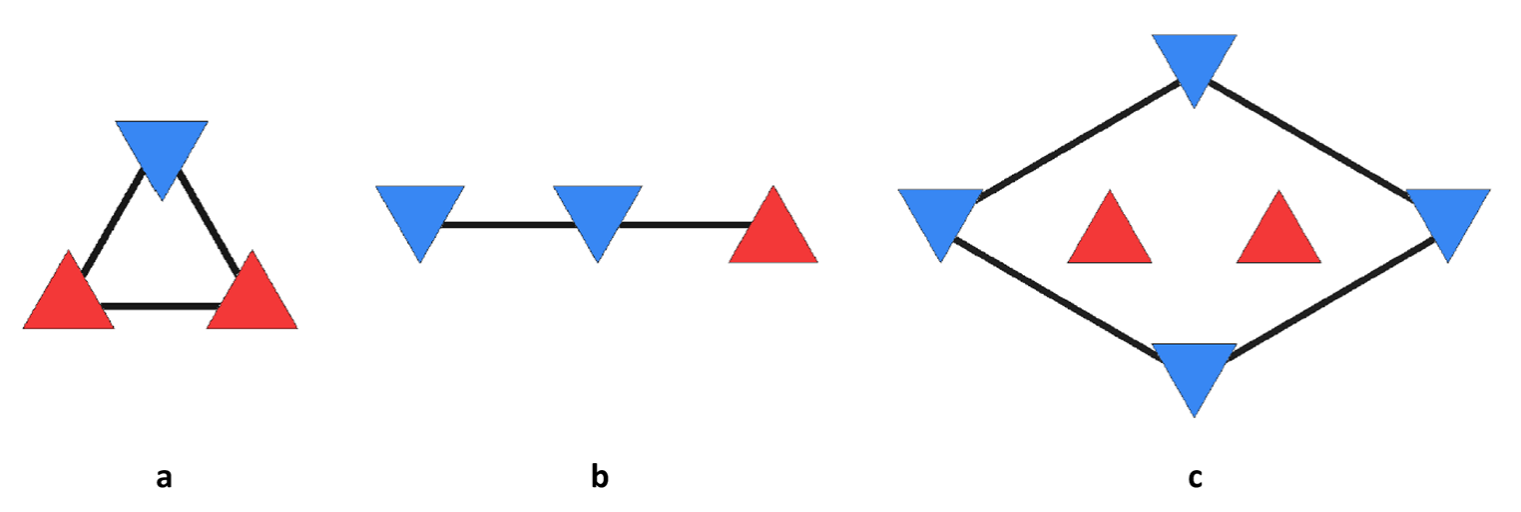
The no-three-alike or superlattice rules. The up (red) and down (blue) triangles denote the two filament rotations. (a) Rule 1 and (b) Rule 2, in which all three filaments do not have the same rotation. (c) A unit cell of the superlattice, in which the rotations at the vertices of the unit cell have the same rotation. The two internal filaments may have like (as here) or unlike rotations

While small patches of superlattice could be seen, it was not clear what the rotations between neighbouring filaments were. Now the task began to find these rotations and whether the superlattice was due to 40^o^ mutual rotations between neighbours to generate a perfect lattice as John had predicted. Evidence had gathered that myosin filaments were not 2-stranded as Huxley had proposed but 3-stranded as proposed by John. Direct inspection of bare region micrographs (Fig. 4b) showed myosin filaments with varying rotations that were possibly 40^o^ rotations. It was a Eureka moment to realise that there were not three rotations (0, 40, 80^o^) but only two, 0 and 180^o^ (the latter is equivalent to 60^o^ due to the triangular symmetry). With the revelation of only two possible rotations, inspection of the distribution of the two rotations revealed that they are only statistically (i.e. not regularly) distributed, and allowed us to derive two rules, the no-three-alike rules, that we called Superlattice Rules 1 and 2, that describe the propensity for like and unlike rotations of neighbouring filaments. Rule 1 states that for any three neighbouring filaments on an elementary triangle, two have the same orientation and the third is different (Fig. 5a). Rule 2 states that for any three neighbouring filaments along a row in the lattice, two filaments have the same rotation and the third is different (Fig. 5b). Essentially, the rules state that three like rotations, either on a triangle or on a line, are unlikely. Note that the rules are not always satisfied, and it was estimated that Rule 1 is violated about 2% of the time and Rule 2 is violated about 25% of the time.

Luther and Squire (1980) used the two rules to build (computationally) synthetic lattices, and the resulting distribution of rotations showed patches of superlattice, i.e. regions where second-nearest neighbour filaments have the same rotation, as is observed in the micrographs. They also constructed optical diffraction patterns from these simulations, and these showed the presence of superlattice reflections in addition to the primary reflections, as is observed in muscle x-ray fibre diffraction patterns. This study therefore established the nature of the superlattice disorder, and the intimate relationship between the superlattice rules, the superlattice, and superlattice diffraction.

## Identification of the superlattice as a frustrated system

The superlattice structure in vertebrate muscle was well-established by the work of Luther and Squire in the 1980s, but new insights began when John described their findings at a Fiber Diffraction Workshop, at McCormick’s Creek State Park, Indiana, USA, in June 1993. His talk resulted in a fruitful collaboration with one of the authors (RPM) in further understanding of the nature of the disorder. I (RPM) had met John previously through both of our work in fiber diffraction, although our main common interest until then had been in the development of digital methods for extracting data from fiber diffraction patterns, a topic that John championed and to which he made important contributions. John’s description of the myosin lattice disorder piqued my interest, as we had been working for some time on models of disorder in crystalline fiber specimens and the effects of the disorder on the diffraction. This was important because polynucleotide fibers, in particular, sometimes exhibit characteristics in their diffraction patterns that indicate specific kinds of disorder in the specimen. To rigorously use the diffraction data for structure determination, these effects need to be accounted for in a quantitatively rigorous manner. Our approach was to derive analytic expressions for the diffraction (e.g. Stroud and Millane (1995)), rather than simply averaging over an ensemble of disordered states. This approach provides more insight into the effects of the disorder and is computationally much more efficient than the latter approach.

The disorder in the myosin filaments described by John mapped precisely to the kinds of specimens we had been considering. The myofibrils are essentially a crystalline array of molecules, with some form of disorder in the disposition of the molecules within the array, and the diffraction is measured from a collection of oriented myofibrils that are randomly rotated about their orientation axis in the muscle fiber. This is exactly identical to the problem of diffraction by a disordered polycrystalline fiber (such as a polynucleotide fiber specially prepared for structure determination by fiber diffraction analysis). The disorder described by John was more complicated than any we had previously considered however, which had involved either uniformly or normally distributed translations or rotations of the molecules away from the positions in an undistorted array. We had, however, considered correlated translations of the molecules at different sites of the lattice, but the correlations were described by a simple exponential correlation field (Stroud and Millane 1996). It was clear to me that the myosin lattice disorder involved a more intricate correlation field of the filament rotations, but the superlattice rules described by John and Pradeep indicated that a suitable description of the correlation field might be obtainable. Furthermore, the correlation field is all that is required to compute the diffraction intensity. The disorder described by John then offered an interesting challenge for us (Millane and co-workers), to characterize the disorder and derive more accurate expressions for diffraction from a muscle fiber. The results should be useful for more rigorous, and more accurate, interpretation of x-ray diffraction data from muscle for structure determination.

Superlattice Rule 1 offers the key insight into the characteristics of the system. This rule implies that nearest neighbour adjacent filaments on a triangle arrange themselves so that the number of adjacent filaments with the same rotation is minimized. It is not possible, of course, to have no adjacent filaments on a triangle with the same rotation, at least two must have the same rotation, simply as a result of the topology of the triangle. There is therefore a preference for *adjacent filaments to have opposite rotations*. It is easy to see that the main observations are a consequence of this simple fact. Rule 1 is a clear consequence. Rule 2 is a simple consequence, since any three like rotations in a row maximizes the number of undesirable interactions, and is therefore unlikely.

**Fig. 6:**
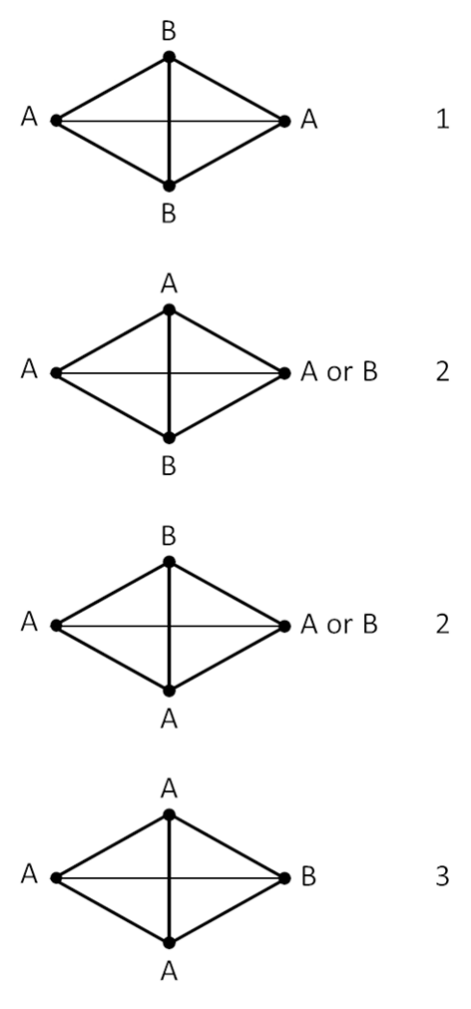
Simple illustration of the preference for like second-nearest-neighbour rotations arising from a preference for unlike adjacent rotations, as described in the text, and leading to the superlattice. Second-nearest neighbour sites are shown at the left and right, and the vertices of the two associated elementary triangles above and below. The four configurations as described in the text are shown, and the number of undesirable interactions for each is shown on the right

The presence of the superlattice is also a direct consequence. The superlattice rhombus joins second-nearest-neighbours of the elementary triangular lattice (Fig. 5c). From the most simplistic point of view, if nearest neighbours prefer unlike rotations, then second-nearest-neighbours will prefer like rotations (Fig. 5c). Overall then, the rotations at the vertices of the superlattice cell (second-nearest neighbours) will tend to be the same. A slightly better approximation is obtained by considering the two elementary triangles most closely involved in second-nearest neighbours, as shown in Fig. 6. Labelling the two rotations as A and B, fixing the rotation at the left site as A, the four possible rotations (A and B) at the center two sites are shown, and the resulting lowest energy rotation at the right hand (second-nearest neighbour) site is shown in the figure. The number of undesirable interactions (adjacent like rotations, or the energy) of each configuration is shown on the right. Inspection of the figure shows that A occurs at the right hand (second-nearest neighbour) site with the lowest energy, A or B occur at the next highest energy, and B occurs at the next highest energy. The same rotation (A) is therefore preferred at the second-nearest neighbour site.

In order to conduct a quantitative analysis of the disorder, parameters are needed to characterize its spatial characteristics. Three parameters were initially chosen for this purpose. These were the frequencies with which the two superlattice rules are violated, denoted *f*_*rv1*_ and *f*_*rv2*_, and the superlattice content, denoted *f*_*s*_. The rule violation frequencies are the fraction of sets of three neighbouring filaments (as shown in Fig. 5a,b) for which the rules are not satisfied. The superlattice content is the fraction of all superlattice rhombi (Fig. 5c) that have the same filament rotation at all four vertices).

It is instructive to make some initial quantitative observations in terms of these parameters. First, the rule violations. As noted by Luther and Squire (1980), Rule 2 is violated more frequently than Rule 1. Referring to Fig. 5a,b, this is unsurprising since a violation of Rule 1 involves three undesirable (adjacent like rotations) interactions whereas a violation of Rule 2 involves only two undesirable interactions. Measurement of the rule violation frequencies from the micrograph data for frog (correctly accounting for the effects of the unknown filament rotations) gave *f*_*rv1*_ = 0.03 and *f*_*rv2*_ = 0.07 (Yoon 2008). Using a simple model of the local environment where the rules apply gives a relationship between *f*_*rv1*_ and *f*_*rv2*_ (see Appendix), and for *f*_*rv1*_ = 0.03 this implies that *f*_*rv2*_ ≈ 0.17. This value is somewhat larger than the observed value of 0.07, but given the approximation of the full lattice by the local environment it is overall consistent with the observations, and provides a quantitative explanation for the higher frequency of Rule 2 violations compared to Rule 1 violations. Second, the superlattice content. For a random distribution of filament rotations, choosing the rotation of one site, the three remaining sites of the superlattice unit cell (Fig. 5c) would have the same rotation each with a probability of one-half. Therefore, the probability of all four sites being the same, i.e. the superlattice content, would be *f*_*s*_ = (1/2)^3^ = 0.125. Measurement of the superlattice content from a frog micrograph gave *f*_*s*_ = 0.36, so the superlattice content is considerably greater than what would be present in a random system.

The above analyses are simple approximations as they are local and ignore the rest of the lattice surrounding the sites considered and that have an effect on the energy of the system. However, they do help to explain the characteristics of the expected distribution of rotations that result from the simple postulate of a preference (lower energy) for unlike nearest neighbour rotations, and that the resulting characteristics concur with what is observed.

We realized early on that the present situation corresponds to a so-called *Ising model* in statistical physics (see e.g. Casquilho and Teixeira (2014)). An Ising model consists of entities, often called spins, on a lattice, and physicists are concerned with studying configurations that emerge on such a system, and associated bulk quantities, as a function of the energetics of the local interactions between nearby spins. A classical two-state spin system on a triangular lattice with only nearest-neighbour interactions, for which unlike nearest-neighbour spins are preferred, i.e. have a lower interaction energy than that for like nearest neighbour spins, is called the *triangular Ising antiferromagnet*, or TIA for short (Wannier 1950). A key characteristic of the TIA is that described above: On an elementary triangular plaquette, it is not possible to simultaneously minimize the energy of all interactions. If two sites have opposite spins (minimum energy), then the total energy is independent of the spin at the third site. This is referred to in physics as *frustration*. The system is frustrated as it cannot decide on the configuration to select to minimize the energy. This particular manifestation is called *geometric frustration* as it results from the geometry, or the topology, of the triangular lattice in this context. An antiferromagnetic system on a square lattice, for example, is not frustrated. As the size of the system increases, from the elementary triangle to a large lattice, the number of minimum energy states increases exponentially with system size. Frustrated systems have many interesting properties and are widely studied in physics (Ramirez 2003). For example, they have a finite zero-point entropy, i.e. there are many minimum energy configurations even at a temperature of absolute zero. The myosin filament lattice appears, therefore, to be a geometrically frustrated system. Although widely studied in physics, this is the first observation of which we are aware of geometric frustration in a native biological system.

We conducted some initial simulations of the superlattice structure by generating lattices based on the rules, similar to that done by (Luther and Squire 1980), but analyzing the results in terms of the rule violations and superlattice content (Millane and Goyal 2000). This basically confirmed the observations of Luther and Squire (1980). Although the observed myosin filament superlattice appears to map to the TIA, we next concerned ourselves with confirming this proposal. This was done by simulating TIA configurations (arrangements of spins or myosin filament orientations) and comparing them with configurations seen in the micrographs.

Minimum energy configurations of the TIA correspond to *ground states*, which occur at a temperature of absolute zero. So-called excited states occur at finite temperatures. Since the muscle fiber is assembled at physiological (non-zero) temperatures, excited state configurations are expected. There is therefore a parameter for the Ising model that is its effective *temperature*. As the temperature increases, the arrangements become more random, rule violations become more frequent, and there is less superlattice.

**Fig. 7:**
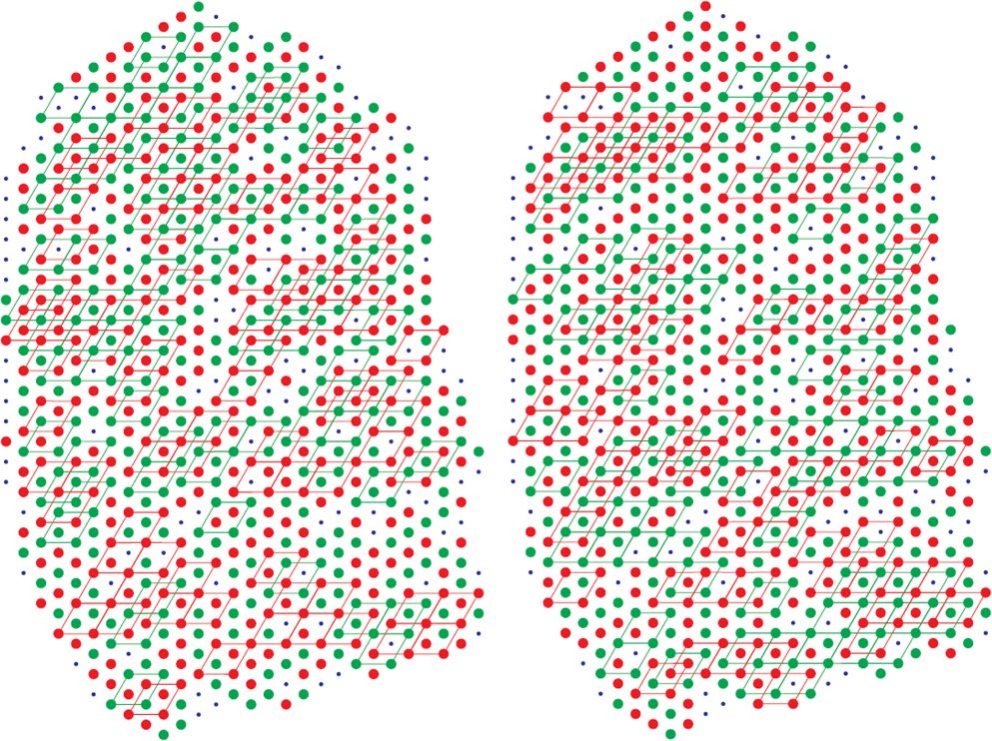
Distribution of myosin filament rotations derived from a micrograph from frog satorius muscle (left) and from a Monte Carlo simulation of the TIA (right) (Yoon 2008). The two rotations are shown by the red and green filled circles, and unknown rotations by the small blue dots. Also shown are the regions of superlattice marked by the rhombi between second-nearest-neighbour sites that all have the same rotation. The two distributions of rotations are not identical, but show similar statistical behavior

Metropolis Monte Carlo simulation, a standard technique for simulating such systems, was used to generate finite temperature configurations for the TIA. The overall distribution of filament rotations obtained from these simulations was reminiscent of those observed in the micrographs. The rule violations and superlattice content were calculated for comparison of the simulation results with the micrograph data. By varying the temperature of the simulation, it was found that there is a particular temperature that gives a good match with the rule violations and superlattice content observed in the micrographs. This gave good support that we were on the right track. An example of the result of such a simulation is shown in Fig. 7. The figure shows the distribution of filament rotations in a full myofibril derived from a micrograph (left) and that from a Monte Carlo simulation (right). Also shown are the superlattice cells in each. The figure shows the similar nature of the disorder in the myofibril and simulation, and the similar degree of superlattice in each.

Rule violations and superlattice content are only three parameters that describe the system and also are not standard parameters. It was decided therefore to use, instead, the spatial correlation function, the correlation between the rotations at two sites, as a function of the separation between the sites, as a more comprehensive function with which to compare models with the data. The correlation function gives a full picture of the second-order statistics of the system. It is also the function that is required to calculate the diffraction intensity. The correlation between two sites would equal 1 if the rotations at the two sites are always the same, would equal − 1 if the rotations at the two sites are always different, would equal zero if the rotations at the two sites are unrelated, and is equal to values in between if there is a preference for like (positive correlation) or unlike (negative correlation) rotations at the two sites.

**Fig. 8:**
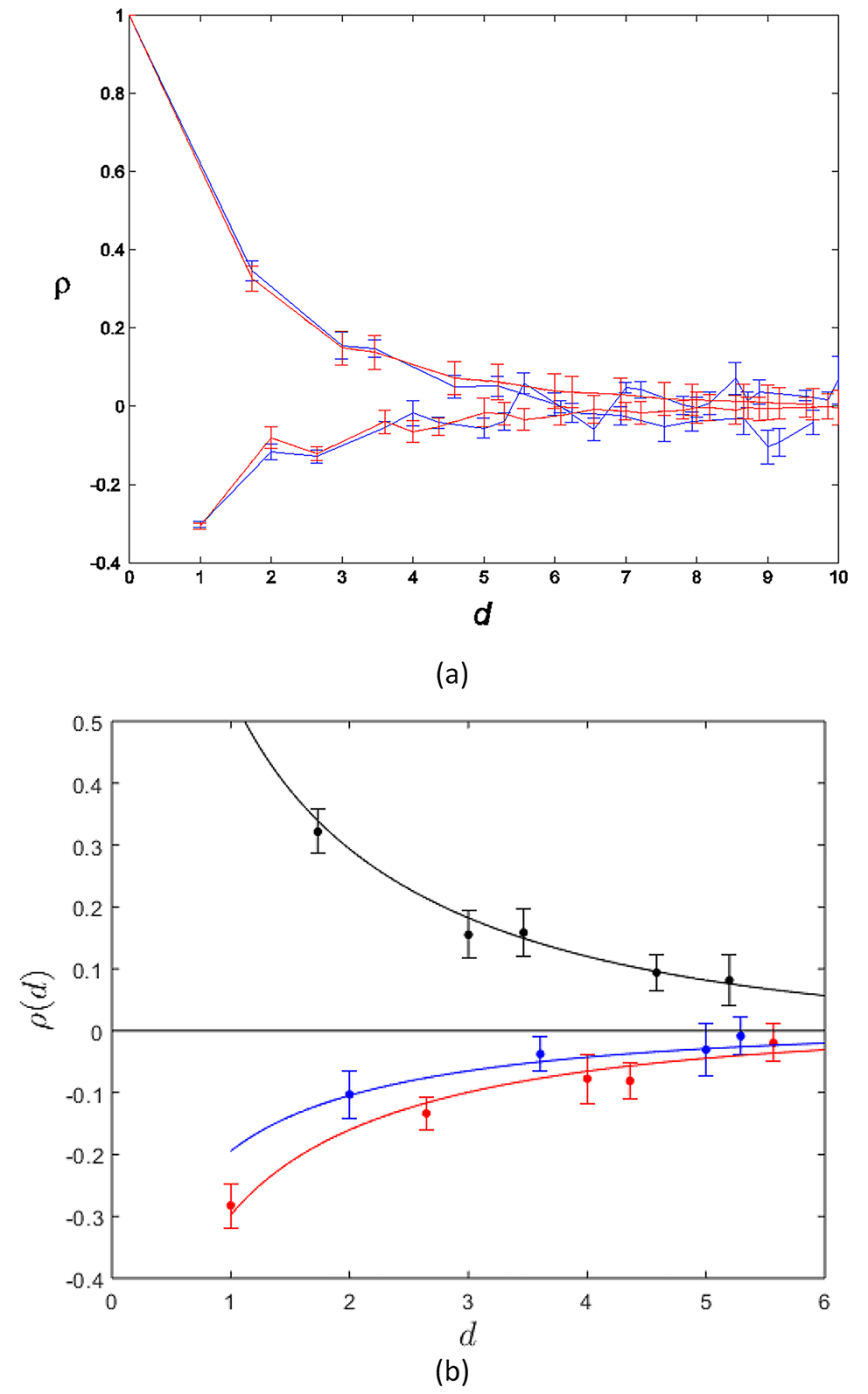
(a) The correlation function of the filament rotations versus the distance between two sites on the myosin lattice measured from a micrograph of frog sartorius muscle (blue points) and calculated from a Monte Carlo simulation of the TIA at the optimum temperature (red points) (Yoon 2008). The error bars show one standard deviation for the measurements and for the Monte Carlo simulation. The points are joined by line segments for clarity. The upper curves show sites on a superlattice and the lower curves for sites off a superlattice. (b) Final comparison of a frog sartorius muscle correlation function (filled circles with bars showing two-standard-errors) and those calculated from the empirical expressions for the TIA at the optimum temperature (curves). The three colours show the three sublattices as described in the text (Millane et al. 2021)

The simulations described above were repeated, and the results analysed in terms of the correlation function. This also produced a good match, at the appropriate temperature, between the correlation function for the TIA and that calculated from the micrographs, out to an intersite spacing of about 8 lattice spacings. An example of the correlation function versus the distance between two sites, derived from a micrograph and from a Monte Carlo TIA simulation at the optimum temperature, is shown in Fig. 8a. Note that the correlation function exists only at the discrete distances that occur between sites on the triangular lattice, but the points are joined by straight lines in the figure for clarity. The correlation function decreases with distance, since the interdependence of the rotations at two sites decreases as the distance between the two sites increases. Note that the correlation function fluctuates rapidly, between positive and negative values, as a function of distance. However, in the figure, we connect the correlation values by two curves, one for pairs of points that belong to a superlattice, and one for pairs of points that do not belong to a superlattice. This shows two smoothly varying functions, with positive correlations for pairs of sites on a superlattice, and negative correlations for pairs of sites not on a superlattice. This emphasizes the superlattice structure, i.e. a tendency for filaments on superlattice sites to have the same orientation, or a positive correlation.

**Fig. 9:**
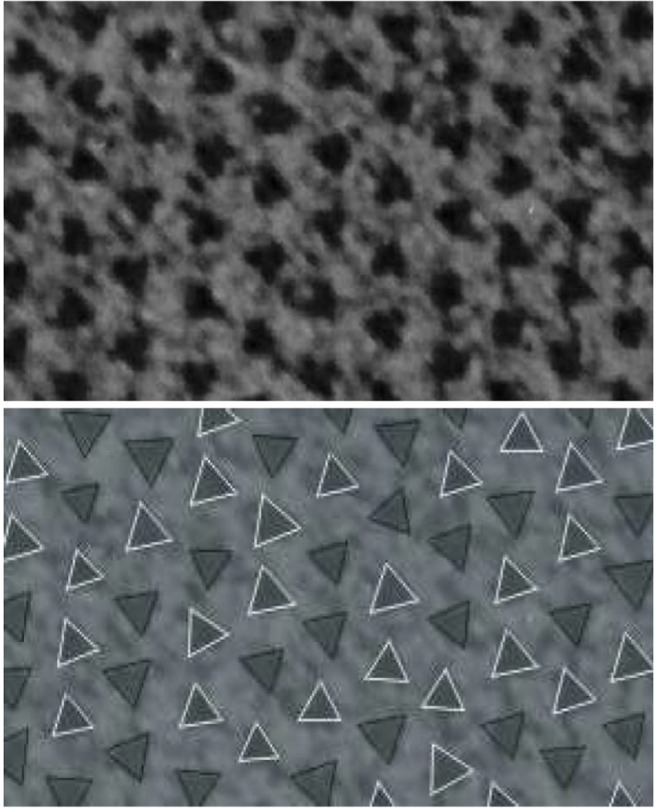
Illustration of analysis of a myosin bare region electron micrograph using the image analysis system (Yoon et al. 2009). A portion of one myofibril in a micrograph through the bare region of frog sartorius muscle (top), and the resulting analysis showing location of the myosin filaments (triangles), estimated rotation of the filaments (indicated by the rotation of the triangles), and classification of the rotations into two populations 60^o^ apart (black and white triangles) (bottom) (Yoon et al. 2009)

The initial calculations described above used filament rotation data derived from Fig. 10 of Luther and Squire (1980). However, their analysis of the micrograph was a little subjective, and also more data from additional micrographs were needed in order to be able to confirm that the TIA is a universal model of the disorder. We therefore developed an image processing system that, using a micrograph as input, automated location of the filaments, and determination and classification of their rotations (Yoon et al. 2009). An example portion of a micrograph, and the location, rotation and classification of the filaments as determined by this system are shown in Fig. 9. This allowed multiple micrographs to be analysed in a convenient, rapid, objective and accurate manner. The system was ultimately used to analyse 15 micrographs from 4 species. Software was developed to use the rotation data to calculate the correlation function from a micrograph and compare it with that from Monte Carlo simulations.

The TIA has been studied extensively in statistical physics and we were keen to see if we could use these results to simplify and provide more insight into our analysis. In particular, there are some analytical results for the correlation function of the TIA (Stephenson 1964). With analytical expressions for the correlation function, the Monte Carlo simulations of the TIA could be avoided completely, practically eliminating most of the computational cost, and providing a more satisfying analysis. While we were initially enthusiastic, it soon became apparent that the available analytical results are very limited. They are accurate only for the “on axis” correlations and at zero temperature. Some approximate expressions are available for finite temperatures, but these are not particularly accurate, and little information is available for the off-axis correlations. Since all of the correlation function (both on- and off-axis) is needed, and for non-zero temperatures, these results were not useful.

However, a useful outcome of this investigation was a better understanding of the so-called *sublattice structure* of the TIA correlation function. Every third site along the primary axis of the lattice corresponds to a site of the superlattice (e.g. the horizontal line through the 4 sites in Fig. 5c). The on-axis zero-temperature correlation function is therefore positive at every third site, decaying with distance. However, the behavior of the correlation at the two intervening sites has a slightly different analytical form, i.e. for distances of 3*n* + 1 versus 3*n* + 2 lattice spacings, where *n* is an integer. This distinction also extends off the primary axis. This means that the correlation behavior is better described on three sublattices, one of which corresponds to the superlattice and the other two which are off the superlattice but are distinct. This is in contrast to the description above that considers two sublattices (i.e. on and off the superlattice). The sublattice structure is described in more detail by Wojtas and Millane (2009) and Millane et al. (2021). The difference is quite subtle, but inspection of Fig. 8a shows a small zig-zag in the correlations in the lower curves, i.e. those off the superlattice, that is diagnostic of this subtle difference. This observation provided more evidence for the validity of the TIA model.

Since accurate analytic expressions for the full TIA correlation function at finite temperatures are not available, we embarked on a project to develop simple, accurate empirical expressions for these functions. The form of the expressions is guided by the on-axis analytical approximations and takes the three sublattices into account, and the expressions were fitted to numerical values derived from Monte Carlo simulations for a range of temperatures. This was very successful and the results are described by Wojtas and Millane (2009). This gave easy to compute correlation functions, without the need for Monte Carlo simulation, to compare with the micrograph data, and these were used for our subsequent studies.

Using all the pieces described above, in our final study we optimized the fit of the empirical expressions for the correlation function for the TIA to the data from 15 micrographs (from four species: frog, shark, polypterus and turtle), finding the temperature for the best agreement, and measured the quality of the fit to the data. The micrograph data and the TIA correlation function are shown in Fig. 8b for one of the frog micrographs. Note the good fit and also that the small difference between the two off-superlattice sublattices (the two lower curves) is replicated in the data. Replication of this subtle feature of the TIA provides further evidence for the TIA, and geometric frustration, as the source of the myosin superlattice disorder. The data and analysis results for all 15 micrographs data are described by Millane et al. (2021). The end result, then, is very strong quantitative evidence that the superlattice disorder in vertebrate muscle is a manifestation of geometric frustration, the TIA, and giving a sound physical basis for its development. The effective temperatures are consistent between all myofibrils and all species except for shark white myotomal muscle which showed higher temperatures. The shark muscle is evidently more disordered, i.e. more random or less evidence of superlattice, than the other muscles. The reason for this is unknown, but it could be related to the observation of super- and simple lattice seen in shark white and red muscle, respectively, as described in the next section.

Our original motivation for this work was to provide a basis for calculating diffraction in the presence of the superlattice disorder. The analytical expressions for the correlation function and the effective temperatures derived from the fits to the micrograph data provide the information to do this, although further theoretical and computational work is required. We have made some progress in this direction in deriving analytical expressions for calculating the two-dimensional diffraction pattern from a two-dimensional array exhibiting the TIA disorder (Yoon and Millane 2014). Using these expressions, simulations of diffraction from such an array show, as might be expected, additional diffuse diffraction between the Bragg reflections of the basic triangular lattice, and an accumulation of diffraction at points reciprocal to the superlattice. There is also modulation of the amplitudes of the Bragg reflections. A more accurate structural result from conventional analysis of the Bragg amplitude data is expected if these modulations are taken into account. Furthermore, with a quantitative description of the amplitudes of the superlattice reflections available, the data set could be expanded to include these data, offering a more robust result. This work needs to be extended to three dimensions and incorporating cylindrical averaging for application to x-ray fiber diffraction studies of muscle specimens. The significance of using a more complete description of the diffraction remains to be seen, but it is certainly likely that this will improve the reliability of structural results.

## Simple and superlattice muscle in vertebrates

Quite early on, John had seen reports in other EM studies that in fish muscle, all the myosin filaments appeared to have a single orientation (Franzini-Armstrong and Porter 1964). Study of the structure of fish slow red and fast white muscle was assigned as the PhD project of Peter Munro. He used electron microscopy to establish that both fast and slow muscles of teleost fish have a single rotation (Luther et al. 1995). We wondered whether there was an evolutionary relationship of the emergence of the simple and superlattice, and undertook an extensive study of vertebrate skeletal muscle looking at diverse species (Luther et al. 1996). First, we established that all tetrapods (mammals, reptiles, birds and amphibians) had superlattice muscle. Then it was a matter of finding examples of fish of different classes. Examples of all the major classes were obtained from different parts of the world with help from scientific colleagues and family and friends. For example, Professor Bo Fernholm brought a live hagfish in a large flask from Stockholm and delivered it to PKL at Heathrow Airport. An African lungfish was obtained from fishermen at Lake Baringo in Kenya. As the majority of the vertebrates sampled showed the superlattice form, it was concluded that the superlattice is the primary arrangement in evolution and the simple-lattice for which teleost fish are the major class, was derived later (Luther et al. 1996). A surprise finding was that in sharks (cartilaginous fish) the lattice was different in fast and slow muscle: white (fast) muscle was superlattice based and slow red muscle was simple lattice. This was a strange and interesting observation, which might be related to the reduced superlattice content seen in shark white myotomal muscle, as described above.

The superlattice-simple lattice story remained a quirk of muscle physiology and was largely forgotten. Then there was a surprise finding in 2019 from the lab of Roger Craig (Ma et al. 2019). Looking at rat fast extensor digitorum longus (EDL) muscle and slow soleus muscles by x-ray diffraction, they noticed that while the fast muscle had the characteristic pattern from tetrapod muscles (Harford and Squire 1986; Huxley and Brown 1967) showing superlattice spots, the pattern from slow soleus muscle was different: it distinctly resembled the fish X-ray pattern, suggesting that the lattice may be simple. They confirmed these findings by EM. This finding for one muscle and one species of tetrapods coupled with the same phenomenon in shark muscle, leads one to suspect that the superlattice/simple lattice arrangement in fast and slow muscles may be common in all vertebrate muscles. This suggests that it may be physiologically important for the function of the muscles. In our previous paper, we proposed that the superlattice arrangement in fast muscle has a more favourable crossbridge distribution around the actin filaments than in simple lattice muscles, hence can produce higher force (described below) (Luther and Squire 2014).

## Discussion

Identification of the TIA as a model for the rotational disorder of the myosin filaments provides a very satisfying physical basis for the observed superlattice in vertebrate muscle, and is a unique known occurrence of geometric frustration in a biological system, as far as we are aware. Although geometric frustration has been observed in physical systems, we are not aware of any in which the frustration is as fully developed as in what we observe here. In this study, non-zero correlations are observed out to eight lattice spacings, whereas in physical systems correlations are generally evident out to only a few lattice spacings.

An explanation of the physical basis of the superlattice disorder is the primary result of this work, together with its potential applications to improve diffraction calculations and thus the precision of the interpretation of such data. There are, however, other aspects of the superlattice disorder that impinge on muscle structure and function, some of which are mentioned in the previous section. Although the data described above are from the bare region, the X-ray diffraction of muscle discussed earlier (Fig. 2a) shows that the superlattice is also present in the overlap region, and thus influences the myosin-actin interactions and the mechanism of contraction. The effective temperature derived from the fits to the TIA allows the interaction energy differences between like and unlike adjacent myosin filaments to be estimated (Millane et al. 2021). The superlattice disorder is expected to be “locked-in” during assembly and cross-linking through the M-band, so that the energy differences are expected to be relevant to connections through the M-band proteins. Although somewhat speculative, this allows the possible degree of sequence differences between proteins of the superlattice and simple lattice muscles to be estimated (Millane et al. 2021).

It has been proposed that the superlattice structure may allow more efficient sharing of actin binding sites by the myosin heads (Luther and Squire 1980). Indeed, there is evidence that slow, fatigue-resistant muscle fibres are associated with the simple lattice and that fast, fatigable fibres are associated with the superlattice (Ma et al. 2019). A greater number of actin–myosin interactions in the superlattice may contribute to the greater force production of fast, fatigable fibres (Luther and Squire 2014). Fast muscle is essential for the rapid movements for escape or pursuit of prey, and clearly slow muscle is ideal for gentle motion. Hence the lattice type may also affect the fibre contractile speed. The lattice type may thus be involved in fine-tuning the mechanical behaviour of muscles.

This study is an interesting example of a synergistic relationship between biological and statistical physics. Mapping a complex biological system to a statistical mechanical model can give fundamental insight into the nature of the biological system, and relate bulk observations (of the pattern of filament rotations seen in electron micrographs) to the fundamental interactions of the components. The study is also a testament to John Squire’s wide-ranging knowledge of muscle structure, his endless curiosity, and his generous and enthusiastic relationships with scientists from a variety of disciplines.

## Appendix: Relationship between the superlattice rules

Here we present a simple model that allows an approximate relationship between the rule violation frequencies to be derived. We consider the environments (or cliques) of the three filaments defining each of the rules (Fig. 5a,b). Note that this is a very simple approximation of the TIA since it does not consider the whole lattice. None-the-less, it gives a simple, approximate understanding of the quantitative nature of the disorder. We consider a simple statistical model for the rotation at each site of the clique, in which the probability of like rotations at two neighbouring sites is given as the quantity *q*. This analysis was conducted with the assistance of Prof. Peter Smith, Victoria University of Wellington.

For the Rule 1 clique, consideration of the symmetry shows that there are only two configurations with distinct probabilities: Either like rotations at three sites or like rotations at two sites. Matching the single-site and two-site marginal probabilities to the probabilities of these two configurations allows the Rule 1 violation probability (frequency) to be related to *q* by

1 $${f}_{rv1}=\frac{3q-1}{2}$$

For the Rule 2 clique, consideration of the symmetry shows that there are only three configurations with distinct probabilities, which take the forms A-A-A, A-A-B, and A-B-A, where A and B denote the two rotations. Matching the one-site and two-site marginal probabilities leaves one degree of freedom for the Rule 2 violation frequency. The Rule 2 violation frequency is then not uniquely defined in terms of *q*, but (for *q* < ½, which is the case here) it is restricted to the range

2 $$0\le {f}_{rv2}\le q$$

Using Eqs. (1) and (2) shows that the two rule violation frequencies are then related by

3 $$0\le {f}_{rv2}\le \frac{2{f}_{rv1}+1}{3}$$

Assuming that *f*_*rv2*_ is uniformly distributed on this range, we can approximate the relationship between the two rule violation frequencies by

4 $${f}_{rv2}\approx \frac{2{f}_{rv1}+1}{6}$$

This gives an approximate relationship between the two rule violation frequencies, with the caveat of the local approximation to the TIA as described above.

## Abbreviations

TIA: Triangular Ising Antiferromagnet
